# *Zataria multiflora* extract reverses lipopolysaccharide-induced anxiety and depression behaviors in rats

**Published:** 2020

**Authors:** Zohreh Arab, Mahmoud Hosseini, Fatemeh Mashayekhi, Akbar Anaeigoudari

**Affiliations:** 1 *Division of Neurocognitive Sciences, Psychiatry and Behavioral Sciences Research Center, Mashhad University of Medical Sciences, Mashhad, Iran*; 2 *Neurogenic Inflammation Research Center, Mashhad University of Medical Sciences, Mashhad, Iran*; 3 *Critical Care Nursing, Faculty of Nursing and Midwifery, Jiroft University of Medical Science, Jiroft, Iran*; 4 *Department of Physiology,* *School of Medicine, Jiroft University of Medical Sciences, Jiroft, Iran*

**Keywords:** Zataria multiflora, Lipopolysaccharide, Anxiety, Depression

## Abstract

**Objective:**

Stressors have an important role in sickness behaviors. We checked the effect of *Zataria multiflora* (ZM) extract against lipopolysaccharide (LPS)-induced anxiety and depression behaviors in rats.

**Materials and Methods:**

Rats were distributed in the following groups (n=10): Control, LPS (1 mg/kg), LPS-ZM50, LPS-ZM100 and LPS-ZM200. LPS was syringed intraperitoneally (ip) 2 hr before performing behavioral tests. LPS-ZM groups were treated with 50, 100 and 200 mg/kg (ip) of ZM extract 30 min before LPS administration. Open field (OF), elevated plus maze (EPM) and forced swimming (FS) tests were done. White blood cell (WBC) was counted in all groups.

**Results:**

In OF, pretreatment with ZM extract augmented the number of lines crossed and traveled distance in central and peripheral areas. The rats treated with ZM extract spent more time in the central zone and less time in the peripheral area compared to the LPS group. In EPM, the number of entries into the open and closed arms and stop time in the open arms in LPS-ZM groups were higher than the LPS group. The stop time in the closed arms of ZM-LPS groups was less than the LPS group. In FS test, swimming and climbing time in groups treated with ZM extract was more than the LPS group while their immobility time was less. WBC count in the LPS-ZM100 and LPS-ZM200 was lower than that of the LPS group.

**Conclusion:**

Based on the results, pretreatment with ZM extract restituted anxiety and depression caused by LPS in rats. This effect of ZM was associated with amelioration of LPS-promoted inflammation.

## Introduction

Worrisome stressors have been found to have a remarkable share in mental sicknesses including anxiety-related disorders and depression–associated disturbances (Normandeau et al., 2018 ▶). Anxiety which threats the heath of people of different ages, is characterized by impairment in mood, madness, panic, excessive activity of the autonomic nervous system frustration, and reduction in learning (O’Brien et al., 2016 ▶; Bedaso and Ayalew, 2019 ▶). Depression also as one of the most earnest disorders threating human health, is characterized by easy relapse, asthenia, and despair (Hankin, 2006 ▶). Inheritance, personality characteristics, environmental factors, decreased level of brain neurotransmitters and illnesses are considered factors causing depression (Gu et al., 2019 ▶). Antidepressant and anxiolytic agents have been proponed to play a basic role in alleviating the symptoms of depression and anxiety through modulating neurotransmitters (Yeung et al., 2018 ▶). It has been reported that treadmill exercise and fluoxetine as a serotonin reuptake inhibitor, can remedy mental disorders such anxiety and depression (Wang et al., 2019 ▶). The use of herbal remedies to attenuate the deleterious effects of depression and anxiety has been also documented (Yeung et al., 2018 ▶). Besides the factors described above, the contribution of brain inflammations to depression and anxiety pathogenesis is also prominent (Brymer et al., 2019 ▶). Previous studies emphasized that the heightened level of pro-inflammatory cytokines followed by stress can result in emergence of depression symptoms (Zheng et al., 2019 ▶; Slavich and Irwin, 2014 ▶). In human models of depression, sleep impairments associated with over activation of transcription factors affecting cytokines genes expression and levels of inflammatory agents such as IL-6, were seen (Miller et al., 2009 ▶). Furthermore, in animal models, the role of bacterial toxic particles including lipopolysaccharide (LPS) in induction of brain inflammations resulting in depression and anxiety was documented (Maes et al., 2012 ▶). For instance, the researchers reported that brain inflammation followed by intracerebroventricular administration of LPS was coupled with a considerable decline in automatic fluctuations in the Y- maze task and an outstanding enhancement in immobility in the suspension test in mice (Ano et al., 2019 ▶). Additionally, peripheral injection of LPS was found to cause sickness behaviors such as immobility, decline in food intake, indifference, and sleep disorders in laboratory animals (Biesmans et al., 2013 ▶; Hart, 1988 ▶). 

*Zataria multiflora* (ZM) is a herbal medicine (Hosseinzadeh et al., 2000 ▶) which is widely known as a painkiller, disinfectant, and antioxidant and an agent with anti- inflammatory properties (Fazeli et al., 2007 ▶; Nakhai et al., 2007 ▶). The extract of ZM was suggested to improve the bad effects of hyperglycemia on diabetic rats through balancing oxidative stress status, diminishing the levels of inflammatory mediators and lowering blood glucose (Mahmoodi et al., 2019 ▶). ZM extract was also shown to lessen inflammatory responses in respiratory system of sensitized guinea pigs (Boskabady and Mahtaj, 2015 ▶). Other pharmacological effects including promotion of β2 adrenergic receptors, and reduction of muscarinic and histamine receptors activity were also attributed to this plant (Kianmehr et al., 2017 ▶). In spite of these reports, the effects of ZM extract on anxiety and depression behaviors have been not studied. Therefore, we were convinced to check the effect of ZM extract on LPS- induced anxiety and depression in rats.

## Materials and Methods

**Animals and groups**

Adult male Wistar rats (n=50) were tested. The animals were kept under standard conditions in terms of lights, humidity and food. The animals were randomly allocated to 5 groups: 1- Control group: the animals received 1 ml/kg of saline; 2- LPS group: the rats were administered with 1 mg/kg of LPS 2 hr before behavioral tests; 3- LPS-ZM50 group, 4- LPS-ZM100 group and 5- LPS-ZM200 group. In groups 3-5, the rats received respectively 50, 100 and 200 mg/kg of ZM extract 30 min before LPS administration. LPS and ZM extract were dissolved in saline and then intraperitoneally administered for 1 week. Experiments on rats were done according to instructions of Ethical Committee of Jiroft University of Medical Sciences (IR.JMU.REC.1398.005). LPS (*E. coli* 055:B5) was bought from Sigma Corporation.

**Preparation of the extract**

ZM was drenched in ethanol (70%) for 72 hr. The solvent was dissociated after leaching the hydro-ethanolic extract of ZM by a special filter. Eventually, the water was evaporated. 

**Behavioral tests**

**Open field**

Locomotor activity and anxiety were examined within five minutes using an open field (OF) arena (Wang et al., 2017 ▶) with dimensions 100×100 cm. Each rat was released in the center of the field and the number of crossing, traveled distance and time spent in the central and peripheral zones, were monitored by a digital camera. Total distance and total crossing were considered indexes of locomotor activity. Meantime, during habituation phase, the rats were released into the apparatus and allowed to search it for 5 min. 

**Elevated plus maze**

We also tested anxiety-related behaviors using the elevated plus maze (EPM) apparatus (Almahozi et al., 2019 ▶). EPM was made of four arms (two open and two closed) 100 cm above of the floor. Homonymous arms were face to face. Before the test day, the rats were accustomed with the apparatus. For this purpose, the animals were left in the apparatus for 5 min. On the test day, each rat was released in the central zone exactly in front of closed arms. The animals were allowed to search the apparatus for 5 min. The number of entry into closed and open arms and stop time in arms were measured using a digital camera. 

**Forced swimming test**

Depression-associated behavior was evaluated by forced swimming (FS) test (Wang et al., 2017 ▶). For this purpose, the animals were put in a glass cylindrical reservoir loaded with water (24-25°C) for 5 min. The measured indicators included immobility, active and climbing time.

**White blood cell count**

The rats were deeply anesthetized by urethane and the blood specimen was taken from retro-orbital sinus. Turk solution was used to prepare the blood sample for counting. The used solution included an equal amount of glacial acetic acid and gentian violet solution 1% as well as 100 ml of distilled water. The total white blood cell (WBC) was carefully measured using a hemocytometer (in a Burker chamber). 

**Statistical analysis**

Data are presented as mean ± SEM. One-way ANOVA followed by Turkey’s *post hoc* was done for analyzing data. A p<0.05 indicated significant differences. 

## Results

**Open field test**

**Figure 1:**
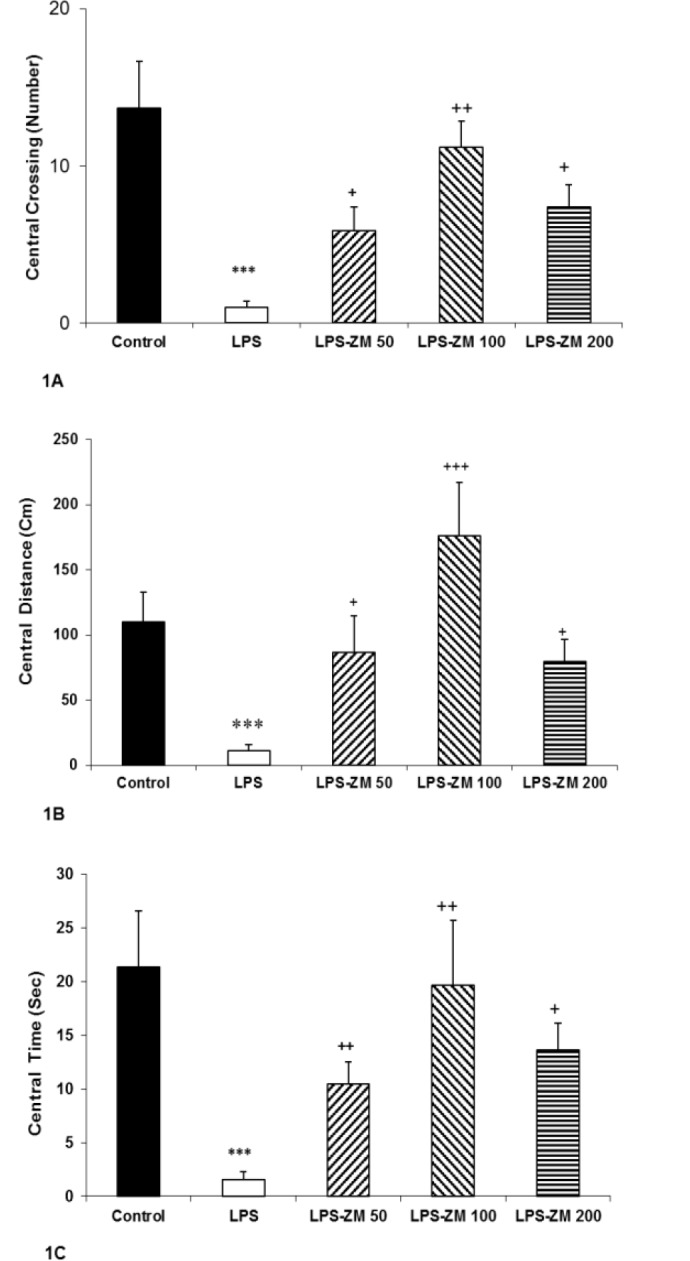
(A) The number of lines crossed, (B) traveled distance and (C) time spent in the central zone in the open field test. Data are presented as mean±SEM (n=10 rats/group). ***p<0.001 indicates significant differences with the control group. ^+^p<0.05, ^++^p<0.01 and ^+++^p<0.001 show significant difference with the LPS group. ^#^p<0.05 and ^##^p<0.01 compared with the control group

**Figure 2:**
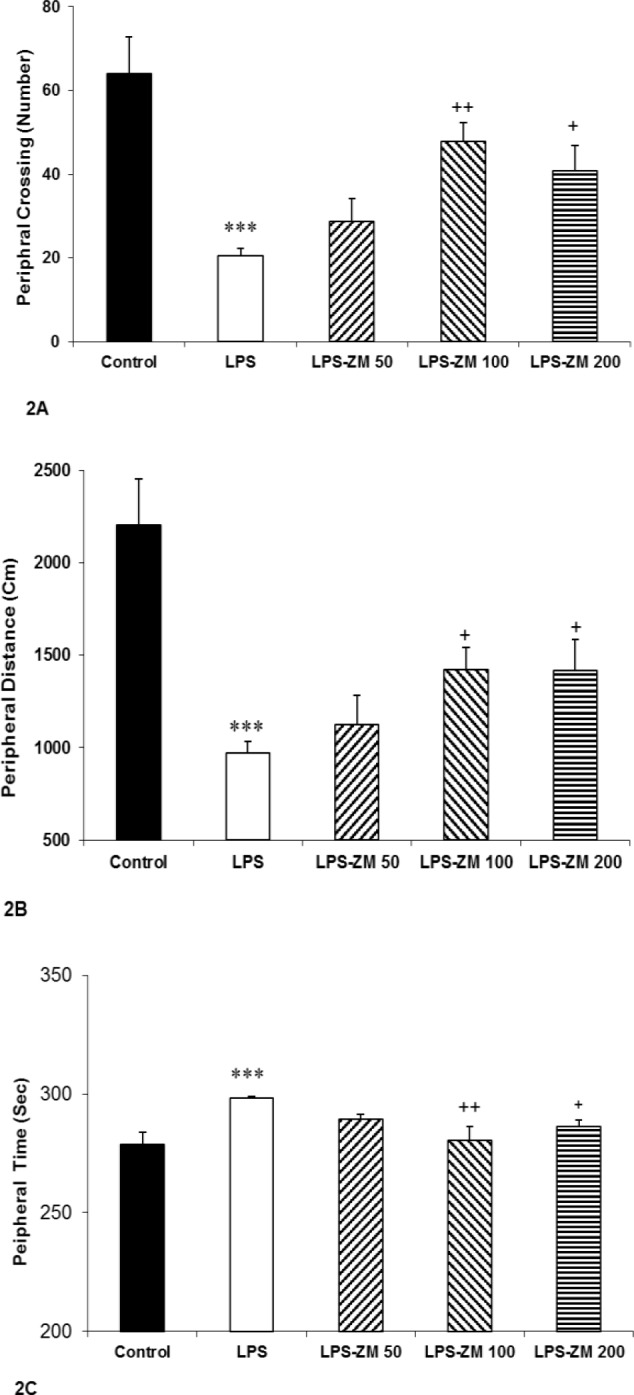
(A) The number of lines crossed, (B) traveled distance and (C) time spent in the peripheral zone in the open field test. Data are presented as mean±SEM (n=10 rats/group). ***p<0.001 and **p<0.01 indicate significant differences with the control group. ^++^p<0.01 and ^+++^p<0.001 show significant differences with the LPS group. ^#^p<0.05 and ^##^p<0.01 and ^###^p<0.001compared with the control group

Based on the OF test results, the animals of LPS group had less central and peripheral crossing compared to the control group (p<0.001). Injection of all three doses of ZM extract increased central and peripheral crossings in LPS-ZM groups compared to the LPS group (p<0.05 and p<0.0, respectively) (Figures 1A and 2A). Also, a remarkable reduction in traveled distance in the central and peripheral area of OF apparatus was seen in rats of the LPS group compared to the control group (p<0.001). Pretreatment with ZM extract resulted in a significant increase in traveled distance in the central and peripheral area in LPS-ZM groups versus LPS group (p<0.05 and p<0.001, respectively) (Figures 1B and 2B). The rats treated with LPS also showed a significant decrease in time spent in the central zone compared to those injected with vehicle (p<0.001). As shown in Figure 1C, injection of ZM extract enhanced the central time in LPS-ZM groups compared to LPS group (p<0.05 to p<0.01). 

**Figure 3:**
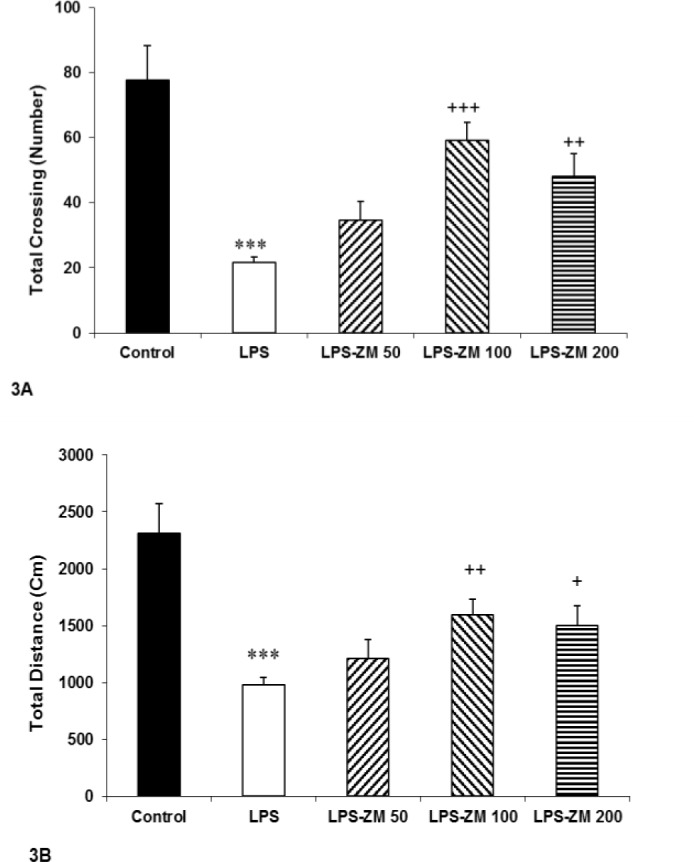
(A) The total number of lines crossed and (B) total traveled distance in the open field test. Data are presented as mean±SEM (n=10 rats/group). ***p<0.001 indicates significant differences with the control group. ^+^p<0.05, ^++^p<0.01 and ^+++^p<0.001 show significant differences with the LPS group. ^#^p<0.05 and ^##^p<0.01 and ^###^p<0.001 in comparison with the control group

Figure 2C indicates that the rats of LPS group spent more time in the peripheral zone than the control group (p<0.01). According to Figure 2C, administration of ZM extract noticeably diminished time spent in the peripheral zone in the LPS-ZM100 and LPS-ZM200 groups compared to the LPS group (p<0.01 and p<0.01, respectively). We also analyzed the data of total crossing and total distance in experimented groups. Findings revealed that the total crossing and total distance of LPS group were considerably lower than those of the control group (p<0.001 for all cases) whereas administration of ZM extract enhanced them in LPS-ZM100 and LPS-ZM200 groups compared to the LPS group (p<0.05 to p<0.001) (Figures 3A and 3B). Comparison of LPS-ZM groups with the control group showed that the animals of LPS-ZM groups crossed lower number of lines in the central and peripheral areas than the control group (p<0.05 to p<0.001) (Figures 1A and 2A). Traveled distance in the peripheral zone (Figure 2B) in groups treated with ZM extract was less than that of the control group (p<0.001). Total crossing and total distance in LPS-ZM groups were also less than than those of the control group (p<0.05 to p<0.001) (Figures 3A and 3B). 

**Elevated plus maze**

**Figure 4:**
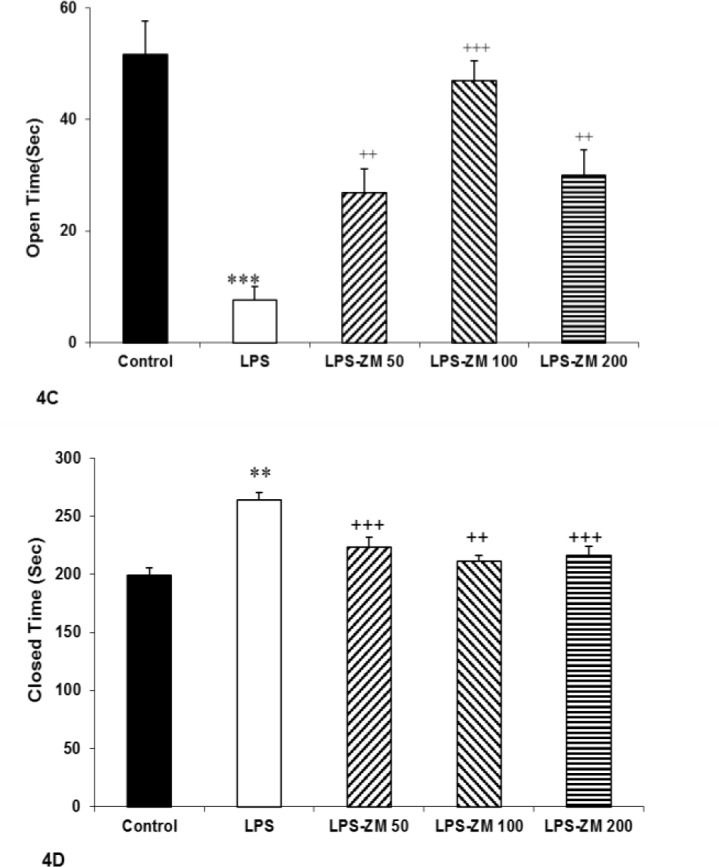
(A) The number of entries into open arms, (B) stop time in open arms, (C) the number of entries into closed arms and (D) stop time in closed arms in the elevated plus maze test. Data are presented as mean±SEM (n=10 rats/group). ***p<0.001 indicates significant differences with the control group. ^+^p<0.05, ^++^p<0.01 and ^+++^p<0.001 show significant differences with the LPS group. ^#^p<0.05, ^##^p<0.01 and ^###^p<0.001compared with the control group

In EPM, the number of entries into the open and closed arms were lower in the LPS group compared to the control group (p<0.001). Increased number of entries into open and closed arms was seen in LPS-ZM50, LPS-ZM100 and LPS-ZM200 groups compared to the LPS group (p<0.01 to p<0.001) (Figures 4A and 4B). The animals treated with LPS also spent less time in the open arm when compared with those injected with saline (p<0.001). We found that time spent in the open arms in LPS-ZM50, LPS-ZM100 and LPS-ZM200 group was higher compared to the LPS group (p<0.01 to p<0.001) (Figure 4C). It was also deduced from Figure 4D that LPS injection meaningfully incremented time spent in the closed arms by rats in the LPS group versus the control group (p<0.01). As shown in Figure 4D, pretreatment with all three doses of ZM extract significantly lessened time spent in the closed arms (p<0.01 to p<0.001). The rats in the LPS-ZM50 and LPS-ZM200 had lower number of entries and spent shorter time in the open arms compared to the control group (p<0.01 to p<0.001) (Figures 4A and 4C). The number of entries to and time spent in closed arms in LPS-ZM50 group were less than those of the control group (p<0.05) (Figures 4B and 4D). 

**Forced swimming test**

**Figure 5:**
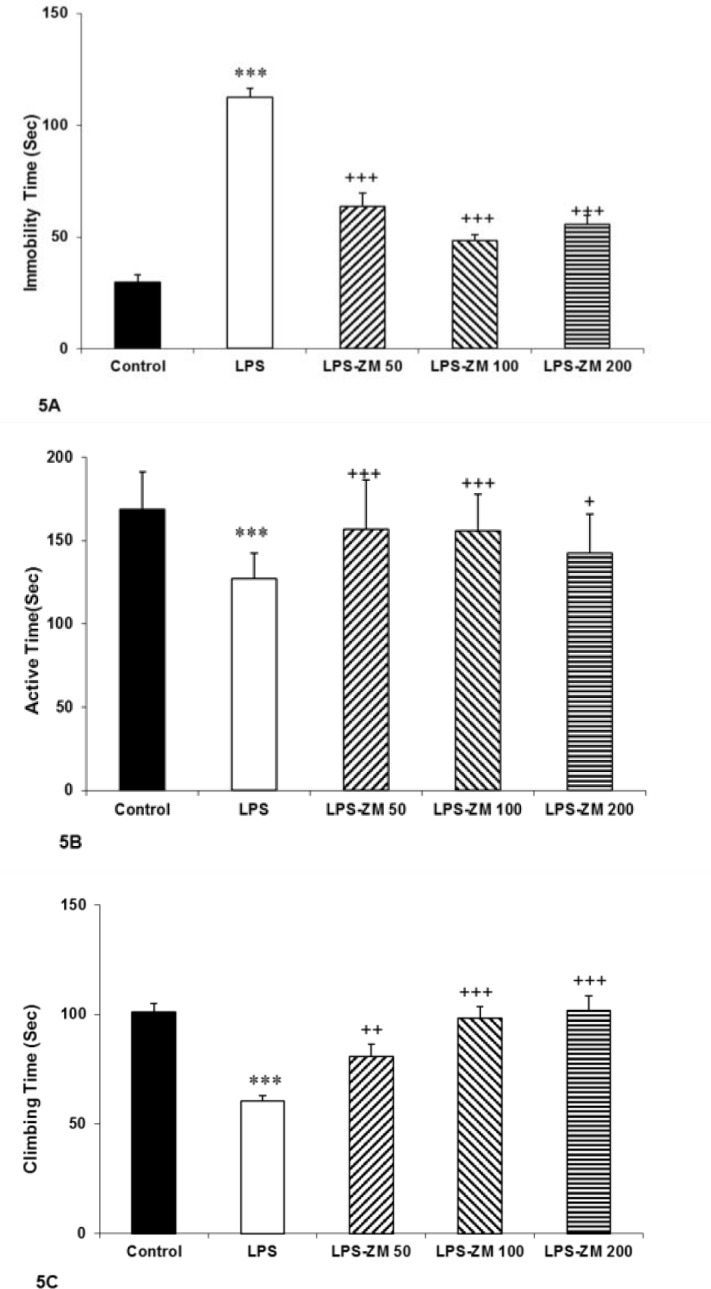
. (A) The immobility time, (B) active time (swimming time) and (C) climbing time in the force swimming test. Data are presented as mean±SEM (n=10 rats/group). ***p<0.001 indicates significant differences with the control group. ^+^p<0.05, ^++^p<0.01 and ^+++^ p<0.001 show significant differences with the LPS group. ^#^p<0.05, ^##^p<0.01 and ^###^p<0.001 as compared with the control group

In this test, we evaluated immobility, active and climbing time in all groups. We concluded from data of FS test that there was a significant augmentation in immobility time but a significant reduction in active time (swimming time) and climbing time in the LPS group compared to the control group (p<0.001). There was a notable reduction in immobility time but a considerable enhancement in swimming and climbing time in rats treated with ZM extract (p<0.05 to p<0.001) (Figures 5A, 5B and 5C). We also found that immobility time in LPS-ZM groups was longer compared to the control group (p<0.001) (Figure 5A). The climbing time in the LPS-ZM50 group was significantly lower than the control group (p<0.001) (Figure 5C). 

**White blood cell count**

**Figure 6:**
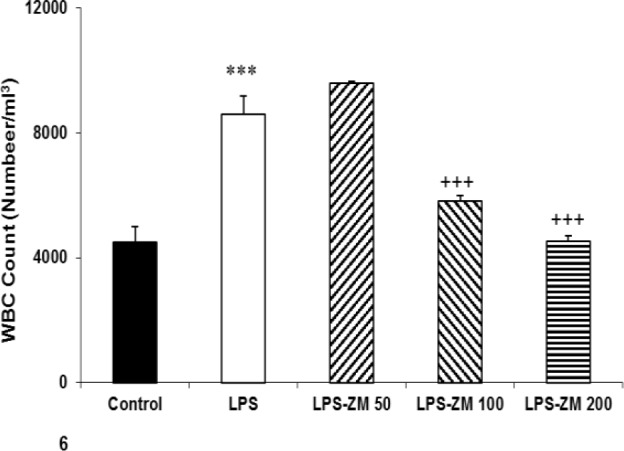
White blood cell count in different groups. Data are presented as mean±SEM (n=10 rats/group). ***p<0.001 indicates significant differences with the control group. ^+++^p<0.001 shows significant difference with the LPS group.^ ###^p<0.001 as compared with the control group

To evaluate the inflammatory responses, we counted white blood cell (WBC) in all groups. Findings illustrated a marked increment in WBC count in the LPS group versus the control group (p<0.001). The WBC count in the LPS-ZM100 and LPS-ZM200 was lower than the LPS group (p<0.001) (Figure 6). The WBC count in LPS-ZM50 was higher than the control group (p<0.001). According to Figure 6, no significant difference was not found in WBC count between LPS-ZM100 and LPS-ZM200 and control group. 

## Discussion

The findings of the present study indicated that depression and anxiety induced by LPS injection were associated with induction of inflammatory responses. Moreover, ZM extract alleviated LPS effects of on depression and anxiety through decreasing inflammatory responses. Depression is a chronic mental disturbance associated with inability, perturbation in thoughts, reduction in interest and anxiety (Bennett and Thomas, 2014 ▶; Gu et al., 2019 ▶). The contribution of Gram-negative bacteria and obtained particles from them such as LPS, to the pathogenesis of depression- related behaviors in both human and animal models has been confirmed (Cordeiro et al., 2019 ▶). It was documented that brain inflammation caused by LPS disturbs mood, activity, cognition and sleep in rodents when it was peripherally injected (Mello et al., 2013 ▶; Dinel et al., 2014 ▶). Similar to previous studies (Almahozi et al., 2019 ▶), we utilized OF and EPM tests to evaluate anxiety–like behaviors and locomotor activity in rats in all groups. In both of the behavior tests, LPS administration disarranged the performance of rats. This finding illustrates that systemic administration of LPS produced the depression and anxiety–like behaviors in rats. In behavioral experiments done in animals, total distance traveled and total number of lines crossed in OF and the number of entries into open and closed arms in EPM, were used as indicators to estimate the locomotor activity (Normandeau et al., 2018 ▶). Based on our results, all these indicators were lower in rats of LPS groups compared to the control group. FS test is also applied as one of behavior techniques to peruse depression–related behaviors (Zhang et al., 2013 ▶; Zheng et al., 2019 ▶). In this test, decrement of immobility time is considered one of basic indicators of behavior disappointment (Cryan et al., 2002 ▶). Our data displayed that injection of LPS increased immobility time and induced disappointment in rats. Beside immobility time, climbing time and active time are also used to assess depressive behavior in FS test. According to the findings of the current research, LPS also decreased the climbing time and active time in rats. 

Raised amount of inflammatory indicators such as IL-6, IL-1β (O’Connor et al., 2005 ▶) and TNF-α (Anisman and Merali, 2002 ▶) accompanied by depression-linked behaviors were seen in animal models. It was also indicated that inflammatory cytokines were increasingly expressed in some of brain areas of rats including hippocampus, hypothalamus and cortex when they are exposed to stressors (Zheng et al., 2019 ▶; Dantzer et al., 2008 ▶). Furthermore, blockage of the activity of transcription factors implicated in inflammatory pathways such as NFκB by anti-depression drugs was shown (Li et al., 2017 ▶). Increased level WBC is also figured out as an index of over-excitation of immune system (Kul’chyns’kyi et al., 2017). There are several reports representing the role of LPS in stimulating immune system cells joined with boosting the numbers of blood leukocytes (Bannerman et al., 2003 ▶). Along with this evidence, we also observed a meaningful increment in WBC count in LPS-exposed rats. This finding is a proof for corroborating this subject that the impact of LPS on depression-like behaviors was presumably mediated via excessive activity of immune system of rats. 

In traditional medicine, ZM was used due to its advantageous effects against radioactive waves (Hosseinimehr et al., 2011 ▶), harmful chemicals (Hosseinimehr et al., 2010 ▶), angiogenesis (Norooznezhad et al., 2017 ▶) and oxidant agents (Sharififar et al., 2007). Ameliorative effects of intraperitoneal administration of ZM oil versus learning and memory dysfunction created by amyloid β (Aβ) in rats, were also reported (Khazdair et al., 2019 ▶). We also deduced that injection of ZM before LPS, suppressed depression–liked behaviors in rats treated with this bacterial particle. In OF test, we found a significant growth in numbers of lines crossed, and traveled distance and time spent in the central and peripheral regions in animals of LPS-ZM groups. In addition, ZM extract noticeably declined the time spent in the peripheral area. We also evaluated the total time and total distance parameters among examined groups. The data showed that administration of ZM extract resulted in a remarkable enhancement in these variables in groups treated with ZM extract. In EPM test, pretreatment with ZM extract meaningfully augmented the tendency to enter the open and closed arms. In addition, we perceived more willingness to stay in the open arms and more avoidance to stop in closed arms in rats treated with ZM extract. It was also detected that treatments that reduce immobility time in FS test, can have antidepressant impacts (Wang et al., 2017 ▶). In line with this finding, we saw a notable decrease in immobility time and a considerable enhancement in swimming time and climbing time in the animals of LPS-ZM groups

Anti-inflammatory and antioxidant effects of ZM on different organs such as the lung (Boskabady and Gholami Mhtaj, 2014 ▶) and colon (Nakhai et al., 2007 ▶) were asserted. Thymol, one of the effective ingredients of ZM, is known to exert ameliorating effects against drugs used in chemotherapy. This effect is attributed to beneficial properties of this compound in modulating oxidative status and inflammatory responses (Arab et al., 2015 ▶). Researchers also reported that ZM extract is able to protect the testicular tissue against oxidative stress caused by cisplatin in mice (Karimi et al., 2018 ▶). Recent reports showed that ZM and its carvacrol could confront toxic effects of acetaminophen on rats hepatocytes (Mohebbati et al., 2018 ▶). It was also demonstrated that ZM extract restored the levels of total WBC, eosinophil, neutrophils, IL-8 and thiol groups in guinea pigs exposed to cigarette smoke as compared to the control group (Boskabady and Mahtaj, 2015 ▶). The results of the current work also indicated that administration of ZM extract diminished the WBC count in LPS-ZM groups compared to the LPS group. Therefore, we assume that the useful impacts of ZM on depression and anxiety behaviors were mediated through modulating inflammatory responses. 

In summary, the results indicated that pretreatment with ZM extract inverted the noxious effects of LPS on depression and anxiety-like behaviors in rats. These effects of ZM are likely to be due to reduction of inflammatory responses induced by LPS injection.
